# YAP1 is essential for tumor growth and is a potential therapeutic target for EGFR-dependent lung adenocarcinomas

**DOI:** 10.18632/oncotarget.19647

**Published:** 2017-07-27

**Authors:** Ting-Fang Lee, Yu-Chi Tseng, Wei-Chin Chang, Yi-Chen Chen, Yu-Rung Kao, Teh-Ying Chou, Chao-Chi Ho, Cheng-Wen Wu

**Affiliations:** ^1^ Institute of Clinical Medicine, National Yang-Ming University, Taipei, Taiwan; ^2^ Institute of Biochemistry and Molecular Biology, National Yang-Ming University, Taipei, Taiwan; ^3^ Department of Pathology, MacKay Memorial Hospital, Taipei, Taiwan; ^4^ Institute of Biomedical Sciences, Academia Sinica, Taipei, Taiwan; ^5^ Department of Pathology, Taipei Veterans General Hospital, Taipei, Taiwan; ^6^ Department of Internal Medicine, National Taiwan University Hospital and National Taiwan University Medical College, Taipei, Taiwan

**Keywords:** YAP1, EGFR mutation, lung adenocarcinoma, TKI-resistance

## Abstract

Epidermal growth factor receptor (EGFR) mutations are found in lung adenocarcinomas leading to tumor cells proliferation and survival. EGFR tyrosine kinase inhibitors (TKIs) that block EGFR activity are effective therapeutics for EGFR-mutant lung adenocarcinoma patients, but TKI-resistance inevitably occurs. The YES-associated protein (YAP1) transcription coactivator has been implicated as an oncogene and is amplified in human cancers and provides tumor cells strong proliferation and survival cues. This study investigated the roles of YAP1 in lung adenocarcinoma by exploring its regulation and functions mediated by EGFR signaling. In this study, we detected a correlation between YAP1 level and EGFR mutation status in lung adenocarcinoma tissues. Using lung adenocarcinoma cell lines, enhanced YAP1 expression and activity mediated by EGFR signaling was detected through enhanced protein stability. A SRC family protein, YES, was involved in EGFR-regulated YAP1 expression and this pathway was crucial for proliferation in EGFR-dependent cells. Small molecules that reduced YAP1 levels by mechanisms bypassing EGFR signaling were effective in reducing viability in EGFR-dependent cells including those with EGFR T790M, the major cause of TKI-resistance. These observations unveiled the significance of YAP1 in EGFR mutant lung adenocarcinomas and identified YAP1 as a promising therapeutic target for EGFR-dependent lung adenocarcinoma patients, including those with EGFR T790M-caused TKI resistance.

## INTRODUCTION

Epidermal growth factor receptor (EGFR) is an oncogenic receptor tyrosine kinase, linking extracellular signals to cellular homeostasis. In EGFR wild-type cells, EGFR signaling is triggered by the binding of its ligands, for example, epidermal growth factor (EGF) and transforming growth factor-α (TGF-α), leading to receptor dimerization and autophosphorylation of its intracellular domain.[1] In EGFR active-mutant cells, EGFR can be constitutively activated in the absence of ligand. Various EGFR alterations have been described in lung cancer. Among these alterations, a small deletion in exon 19 and a point mutation, L858R, in exon 21 are the most common.[2] EGFR activation generates potent growth and survival signals that enable tumors to grow.

EGFR-activating mutations are found with high frequency of 40 to 55 % lung adenocarcinomas in East Asia [3–5] in which tumor cells are dependent on EGFR signaling. The use of tyrosine kinase inhibitors (TKIs), such as gefitinib and erlotinib, in EGFR-mutant lung adenocarcinoma patients successfully causes tumor regression and prolonged patient survival; [6] however, drug resistance and tumor relapse eventually occur. The major cause of drug resistance is the T790M mutation, a secondary EGFR mutation that disables TKI function and allows tumor cells to continue to rely on EGFR.[7] Recently, second-generation TKIs, afatinib, have been developed to treat lung adenocarcinoma with EGFR-activating mutations,[8] however, the response rate to T790M was not satisfactory. Next-generation EGFR inhibitor, AZD9291, has been approved to fight against this most frequent cause of TKI-resistance, T790M. Nevertheless, acquired EGFR C797S mutation has been reported.[9]

YES-associated protein (YAP1), a transcriptional coactivator, is a major determinant of tissue growth and organ size.[10, 11] YAP1 mutation has recently been reported to be associated with familial lung adenocarcinoma.[12] Elevated YAP1 expression and nuclear localization have been noted in different cancer types, including lung cancer.[13–15] Through interactions with the transcription factor TEAD, YAP1 promotes tissue growth through the simultaneous induction of cell proliferation and inhibition of apoptosis. YAP1 is well known to be regulated by Hippo signaling and also other Hippo-independently pathways by mechanical and architectural cues, such as cytoskeletal tension and cell shape. [16, 17]

Because EGFR is the predominate driving oncogene in lung adenocarcinoma and because of the emerging roles of YAP1 in lung cancer, here we are interested in investigating the interactions between these two significant growth signals, anticipating to identify an alternative target that is downstream of EGFR. In this study, we identified YAP1 as an important pathway mediated by EGFR signaling that regulates cell growth. We suggest that through bypassing the driving oncogene EGFR itself, the YAP1 pathway can act as a promising alternative therapeutic target for lung adenocarcinoma patients with EGFR-dependency including those with EGFR T790M gatekeeper mutation.

## RESULTS

### YAP1 expression correlates to EGFR active mutation

**Figure 1:**
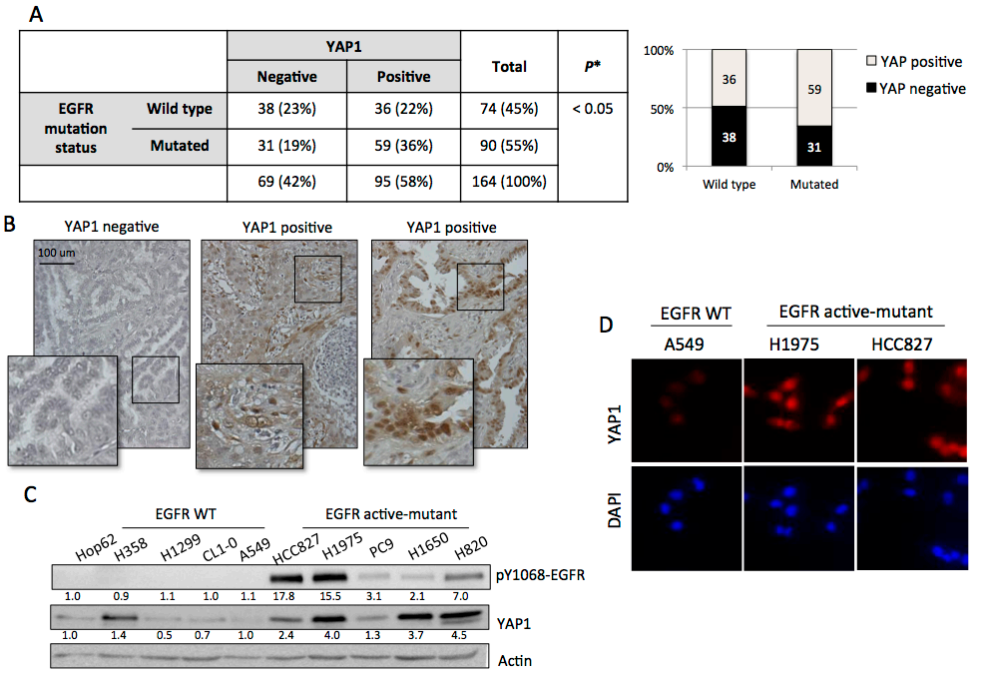
YAP1 expression correlated to EGFR mutation status **A.** YAP1 expression and EGFR mutation status were analyzed in 164 cases of NSCLC tissue. Positive YAP1 staining was observed in 58% (95/164) cases while EGFR active-mutation detected in 55% (90/164). YAP1 expression was correlated with EGFR mutation status (*p* = 0.03). Comparison of EGFR mutation status vs YAP1 positivity was analyzed by Fisher's exact test. **B.** Representative images showed YAP1 (brown staining) expression in lung adenocarcinoma tissue. **C.** After 4h serum starvation, ten lung adenocarcinoma cell lines: five EGFR wild-type and five EGFR active-mutant, were collected. EGFR mutant cell lines showed endogenous EGFR phosphorylation and upregulated YAP1 expression. **D.** The EGFR active-mutant lung adenocarcinoma cell lines H1975 and HCC827 had stronger YAP1 signal and nuclear localization compared to EGFR wild type A549 detected by immunocytostaining.

To identify if YAP1 is a possible target for EGFR-dependent lung adenocarcinomas, we investigated the correlation between EGFR mutation status and YAP1 expression in 164 cases of NSCLC tissue. YAP1 immunoreactivity was graded as negative and positive by a pathologist. Positive YAP1 staining was observed in 58% (95/164) cases. The EGFR mutation status was carried out using PCR method [18]. Wild-type EGFR was detected in 45% (74/164) cases and active-mutant EGFR detected in 55% (90/164) (Figure 1A). Representative images showed YAP1 negative and positive expression results (Figure 1B). As shown in Figure 1A, YAP1 expression was correlated with EGFR mutation status (*p* = 0.03). Compared to the EGFR wild-type group, the EGFR mutant group has higher YAP1 positive rate. This result suggested that active-mutant EGFR promoted YAP1 expression. To confirm this finding *in vitro*, five EGFR wild-type and five EGFR active-mutant lung adenocarcinoma cell lines were collected. Immunoblotting revealed constitutively expressed EGFR phosphorylation signal in EGFR mutant cells as well as upregulated YAP1 levels compared to EGFR wild-type cell lines (Figure 1C). Immunocytochemistry also demonstrated enhanced and nuclear localized YAP1 expression in EGFR mutant cell lines (Figure 1D). Here we demonstrated a correlation between EGFR mutation and YAP1 expression using both human tissues and cancer cell lines.

### EGFR signaling promotes YAP1 expression and activation

**Figure 2:**
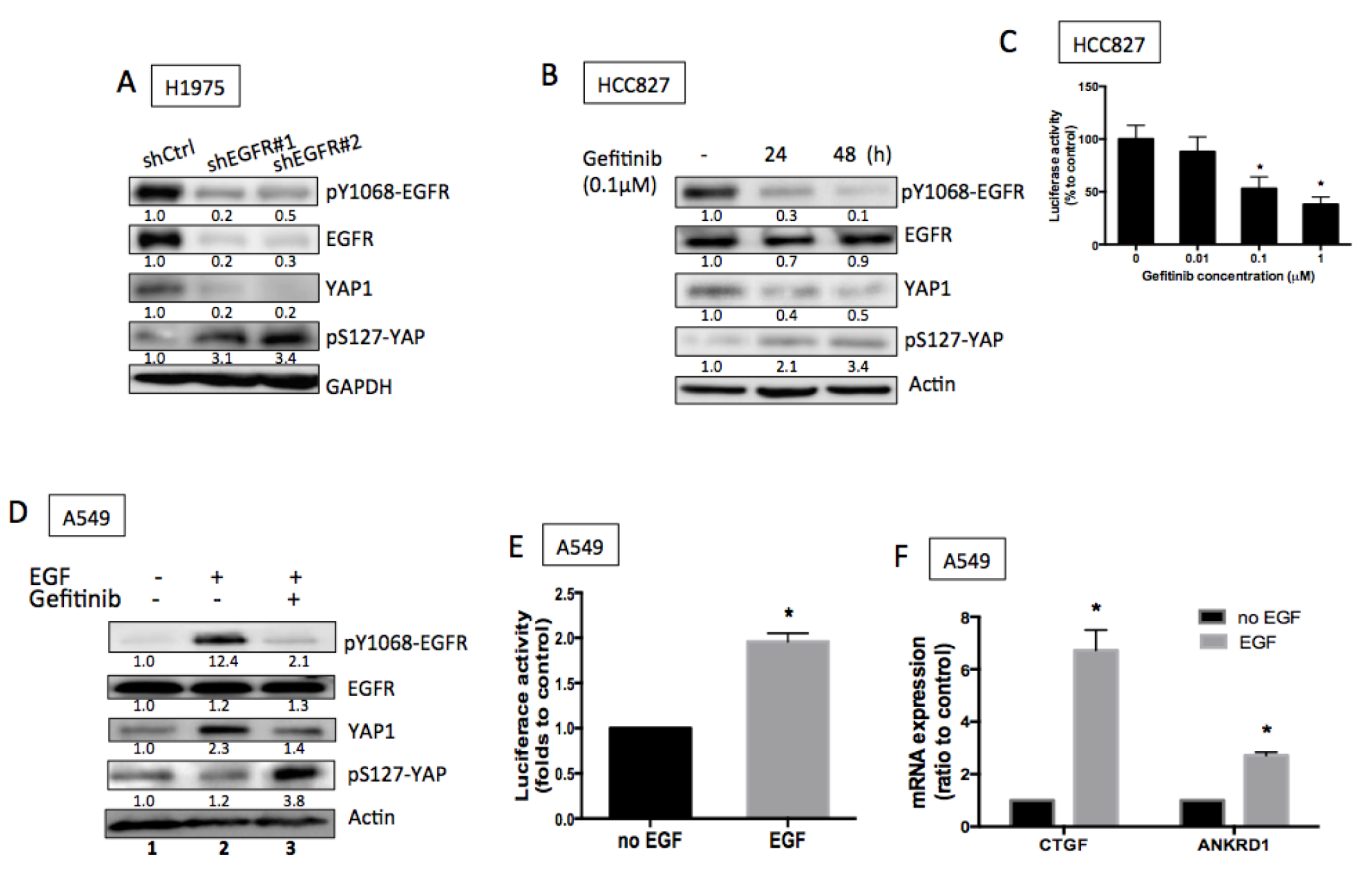
EGFR signaling promoted YAP1 expression and activity **A.** Knocking down EGFR using shRNAs reduced EGFR phosphorylation and total YAP1 expression. **B.** Gefitinib, an EGFR TKI, blocked EGFR phosphorylation and reduced YAP1 protein levels. **C.** 8XGTIIC luciferase activity assays demonstrated reduced YAP1 activity in the presence of gefitinib in HCC827 cells. The error bars represent the S.E. of 3 independent experiments. *P < 0.05. **D.** EGF (1 h) triggered an increase of YAP1 in A549 cells while gefitinib blocked both EGFR phosphorylation and YAP1 level. **E.** Increased luciferase activity triggered by EGF (2 h) indicated increased YAP1 activity. The data are presented as mean±S.E. of three independent experiments. *P < 0.05. **F.** Upregulated mRNA expressions of *CTGF* and *ANKRD1*, the TEAD target genes, were stimulated by EGF (2 h). The error bars represent the S.E. of four independent experiments. *P < 0.05.

To further identify the regulation of YAP1 by EGFR signaling, we suppressed EGFR activity using either shEGFR or EGFR inhibitors in EGFR active-mutant cell lines. Knocking down EGFR in H1975 cells exhibited suppressed EGFR phosphorylation and suppressed total YAP1 protein levels together with enhanced phospho-YAP (S127) expression (Figure 2A). Reduced total YAP1 demonstrated by immunostaining in the presence of shEGFR or EGFR inhibitor (Figures S1A & B). Gefitinib, an EGFR inhibitor, blocked EGFR phosphorylation, reduced total YAP1 expression and upregulated phospho-YAP expression in HCC827 cells (Figure 2B). To detect whether total YAP1 protein levels correlate to its activity, we used a synthetic YAP1-responsive luciferase reporter, 8XGTIIC-Luc containing multimerized responsive elements of TEAD, as a read-out of the transcriptional function. Gefitinib concentration-dependently reduced the luciferase activity, confirming reduced YAP1 activity by blocking EGFR signaling in EGFR mutant cells (Figure 2C). Also, down-regulated *CTGF* and *ANKRD1* detected in the presence of EGFR inhibition (Figure S1C & D).

Knowing that reduced EGFR activity was in direct correlation to reduced YAP1 expression and activity, we next investigated whether upregulated EGFR activity promoted YAP1 expression in EGFR wild-type cells in the presence of EGF. When we treated EGFR wild-type A549 cells with EGF, a remarkable increase in YAP1 protein level was triggered along with EGFR phosphorylation (Figure 2D, lanes 1 & 2). This phenomenon was also detected in H1299, another EGFR wild-type line (Figures S2A left). Gefitinib effectively blocked EGFR phosphorylation and decreased YAP1 protein levels (Figure 2D, lanes 2 & 3). EGFR internalization was detected shortly after EGF stimulation along with an increase in YAP1 expression (Figure S2B). Two EGFR ligands, EGF and TGF-α, both induced EGFR phosphorylation and increased YAP1 protein levels (Figures S2C and D). EGF treatment (2 h) induced expression of transfected 8XGTIIC-Luc, indicating increased YAP1 activity (Figure 2E). Along with enhanced YAP1 activity, the target genes *CTGF* and *ANKRD1* were also upregulated (Figure 2F & S2A right) in the presence of EGF (2 h). Taken together, our data indicate that EGFR signaling increases YAP1 protein levels and activity.

Worth mentioning that, the expression of YAP1 is well known to be regulated by cell density [19] and the cell lines we used were not exempt: YAP1 expression was dramatically reduced in densely cultured cells in A549 and H1975 cells (Figure S2E). And thus, cells for all the *in vitro* experiments in this study were done in sub-confluent condition to prevent the strong contact inhibition effect.

### EGFR signaling promotes YAP1 protein expression through enhanced protein stabilization

**Figure 3:**
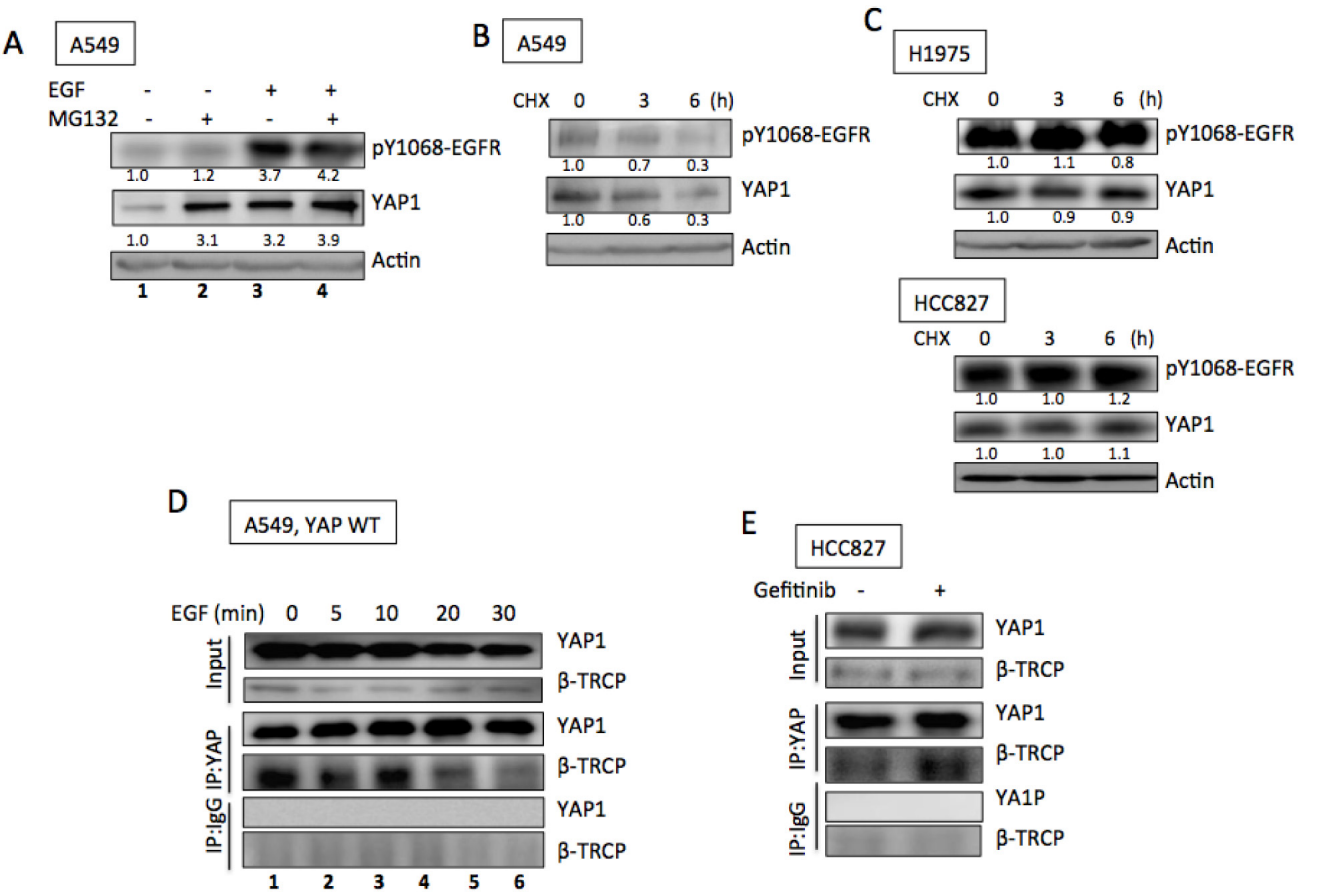
EGFR promoted YAP1 stabilization **A.** MG132 (10 μM, 3 h) induced YAP1 accumulation in A549 cells in the absence of EGF (lanes 1 and 2); while no further increase of YAP1 level induced by MG132 (3 h) in the presence of EGF (1 h) (lanes 3 and 4). CHX (20 μg/ml) treatment caused decreased YAP1 levels in A549 cells **B.** while YAP1 levels maintained in H1975 and HCC827 cells **C.** with CHX treatment. **D.** By immunoprecipitating YAP1, its binding with β-TRCP was detected in A549 cells. EGF treatment reduced the binding between YAP1 and β-TRCP. **E.** In the presence of gefitinib (0.1 μM, 24 h), enhanced YAP1/β-TRCP binding was detected in HCC827 cells.

Because *YAP1* mRNA levels were not affected by EGF treatment in A549 cells or by EGFR knockdown in H1975 cells (Figures S3A and B) and because YAP1 has been reported to be an short-lived protein, [20] these data suggest that EGFR signaling increased YAP1 protein levels by enhancing protein stabilization. Next, we investigated whether EGFR signaling promoted YAP1 expression by increasing its protein stability. YAP1 is known to be degraded via the proteasome degradation pathway, and MG132, a proteasome inhibitor, is used to inhibit YAP1 degradation. In the absence of EGF, treating A549 cells with MG132 caused YAP1 protein accumulation (Figure 3A, lanes 1 & 2). However, in the presence of EGF stimulation, MG132 could not further enhance YAP1 protein levels (Figure 3A, lanes 3 & 4), suggesting that EGF and MG132 induced redundant signals regulating YAP1 protein degradation and EGFR signaling prevented YAP1 degradation by the proteasome pathway. On the other hand, CHX, a chemical that blocks protein translation, effectively reduced YAP1 protein levels in A549 cells (Figure 3B), while the EGFR active-mutant H1975 and HCC827 cells maintained YAP1 levels in the presence of CHX (Figure 3C). These results supported the hypothesis that YAP1 degradation is suppressed in the presence of EGFR signaling.

Because YAP1 is an short-lived protein degraded by the proteasome pathway through β-TRCP binding followed by ubiquitination, we further confirmed the role of the EGFR pathway in YAP1 degradation by testing the binding between YAP1 and β-TRCP. We expressed YAP1 in A549 cells and immunoprecipitated YAP1 protein. In the absence of EGF, strong β-TRCP binding was detected by immunoblotting (Figure 3D, lane 1), suggesting the presence of YAP1 degradation. Upon EGF treatment, decreased β-TRCP binding was observed within 30 min (Figure 3D, lanes 2-5), indicating a reduced amount of YAP1 degradation in the presence of EGFR signaling that leads to enhanced YAP1 level. On the other hand, in the presence of gefitinib, HCC827 showed enhanced YAP1/β-TRCP binding (Figure 3E), suggesting promoted YAP1 degradation induced by EGFR inhibitor. The above results confirmed that EGFR signaling enhanced YAP1 expression by promoting protein stability.

### YAP1 is essential for cell proliferation and survival in EGFR-dependent cells

**Figure 4:**
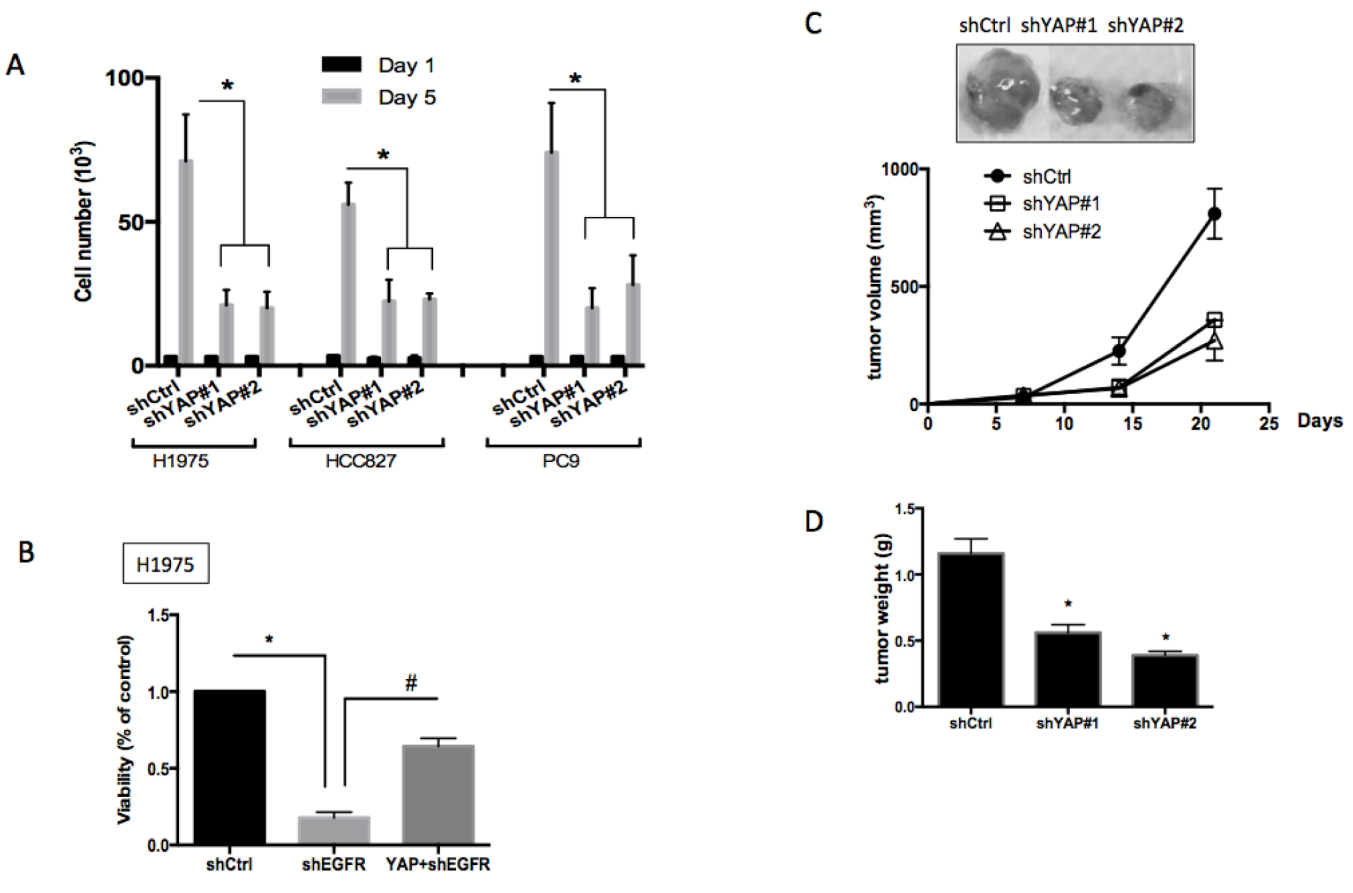
Roles of YAP1 in cell proliferation, survival and tumor growth **A.** Cell proliferation assay for H1975, HCC827 or PC9 cells knocking down scramble control or YAP1. Cells knocking down YAP1 showed significantly reduced proliferation. The error bars represent the S.E. of four independent experiments. *P < 0.05. **B.** Knocking down EGFR in the EGFR-dependent H1975 cells reduced cell viability and forced YAP1 expression rescued loss-of-EGFR-caused viability loss. The error bar represents the S.E. (*n* = 4). *P < 0.05 control compared with shEGFR, and #*P* < 0.05 shEGFR compared with YAP1+shEGFR. Subcutaneous injection of H1975 cells knocking down scramble control or YAP1 into nude mice. Reduced tumor size **C.** and reduced tumor weight **D.** was detected in YAP1 knockdown groups. The data are presented as the mean±S.E. (*n* = 6 per group).*P < 0.05.

Finding that EGFR signaling triggers an increase in YAP1 protein expression and its activity, we next focused on understanding the biological roles of YAP1 in EGFR-dependent lung cancer cells. Knocking down YAP1 in H1975, HCC827 or PC9 cells resulted in significant inhibition of cell proliferation (Figure 4A), indicating that YAP1 plays a role in the growth of EGFR-mutant tumor cells. Reduced YAP1 protein levels in shYAP1 groups were shown (Figure S3C-E). Upon knocking down EGFR in EGFR-dependent H1975 cells, the loss of EGFR signaling significant reduced cell viability, which could be rescued by overexpressing YAP1 (Figure 4B). This observation demonstrated that forced YAP1 expression can rescue cancer cells with EGFR oncogene dependency from EGFR suppression, suggesting that YAP1 plays a survival role in lung adenocarcinoma.

A subcutaneous tumor xenograft model was used to further demonstrate whether YAP1 is required for tumor growth *in vivo*. We injected H1975 cells expressing one of two different YAP1 shRNAs or scramble control subcutaneously into nude mice. Three weeks after tumor cell injection, tumors with diminished YAP1 expression exhibited a substantially smaller volume (Figure 4C) and lower weight (Figure 4D). The xenograft tumor results indicated that YAP1 was essential for tumor growth in EGFR-dependent lung adenocarcinomas *in vivo*.

### YES is involved in EGFR-mediated YAP1 stabilization and function

**Figure 5:**
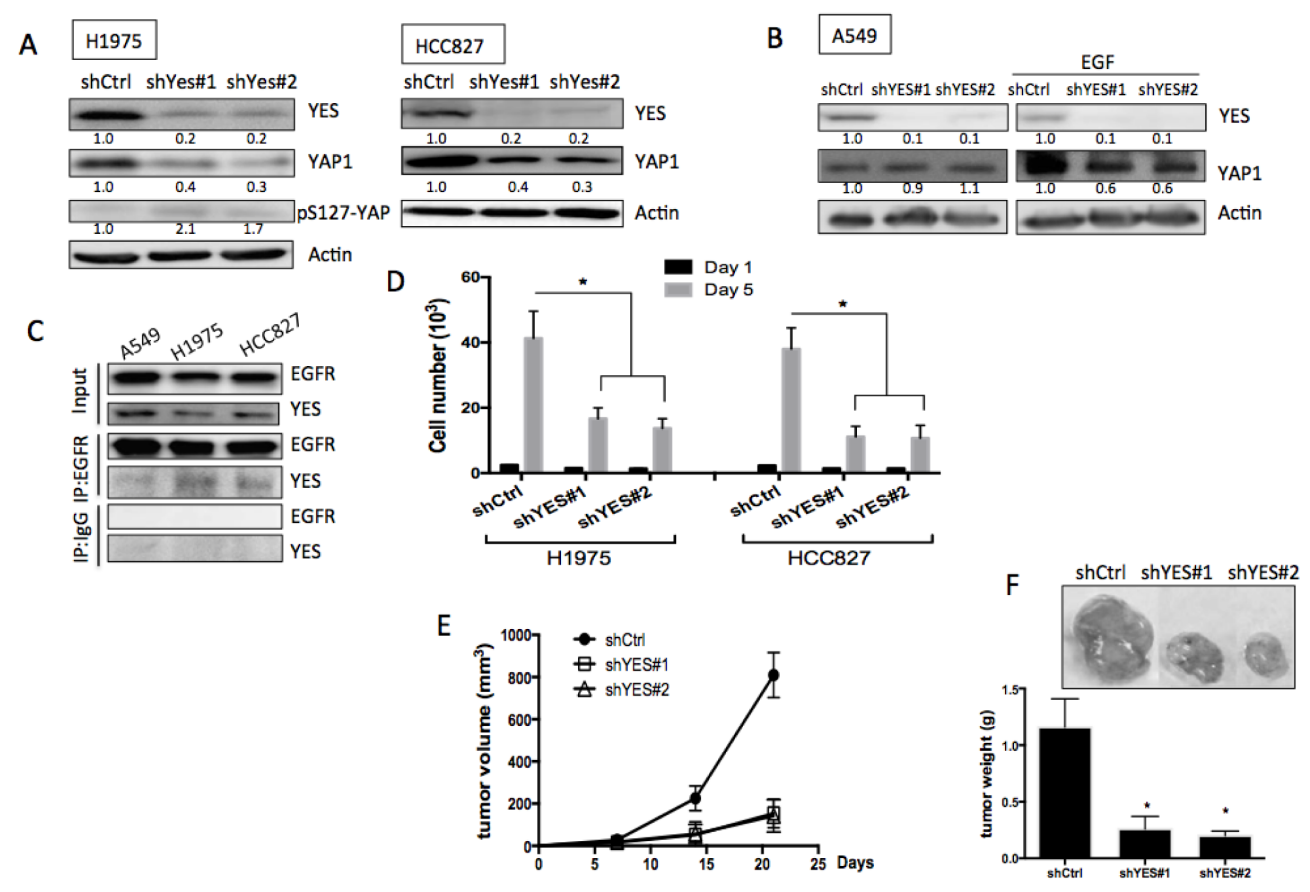
Role of YES in EGFR-mediated YAP1 expression and function **A.** Knockdowns of YES in EGFR mutant cell lines, H1975 and HCC827 caused reduced YAP1 expression. **B.** Knockdowns of YES did not reduce YAP1 level in A549 (left panel). EGF (1 h) triggered YAP1 expression was diminished in YES knockdown groups (right panel). **C.** Immunoprecipitated EGFR can detect YES expression in H1975 cells but not in A549. **D.** Knockdowns of YES caused decreased cell proliferation in H1975 cells. The error bars represent the S.E. of three independent experiments. *P < 0.05. Subcutaneous tumor xenograft model showed knockdowns of YES in H1975 cells reduced tumor size **E.** and tumor weight **F.** The data are presented as the mean±S.E. (*n* = 6 per group).*P < 0.05.

YAP1 was originally identified as a YES-associated protein. We observed that the SRC family protein YES was essential for EGFR-mediated YAP1 protein stabilization. Knockdowns of YES in EGFR-mutant H1975 or HCC827 cells, but not in wild-type EGFR A549 cells, decreased YAP1 levels (Figures 5A and 5B, left). EGF was unable to trigger an increase in YAP1 levels in A549 cells with YES knockdown (Figure 5B, right). Reduced CTGF and ANKRD1 mRNA expression detected in YES knockdowns in EGFR-mutant H1975 and HCC827 cells (Figure S4A), suggesting reduced YAP1 activity. By immunoprecipitating EGFR, the binding with YES was detected in H1975 and HCC827 but not in A549 cells (Figure 5C), suggesting an interaction between EGFR and YES in EGFR-mutant cells. Furthermore, by immunoprecipitating YAP1, its binding with YES was detected (Figure S4B), confirming the interaction between YES and YAP1.

We also tested the possibility of forming an EGFR-YES-YAP1 complex in lung cancer cells. By immunoprecipiting YAP1 with EGFR blotting, or vise versus, the binding between EGFR and YAP1 was not detected (Figures S4B and C). Immunostaining result also cannot identify the colocolization between EGFR and YAP1 (Figure S4D). These results suggest that YES acts as a mediator, transmitting EGFR signal to YAP1, and thus, limiting the direct contact between EGFR and YAP1. Moreover, SRC that is known to interact with EGFR [21, 22] has recently been reported to regulate YAP1 stability. [23] Here we also detected reduced YAP1 expression in SRC knockdowns in H1975 cells (Figure S4E), suggesting a regulation of YAP1 by SRC.

We next assessed whether YES is involved in the biological functions of EGFR-mutant lung cancer cells. In proliferation assays, significantly reduced growth was detected in H1975 cells with YES knockdowns but not in A549 with YES knockdowns (Figure 5D & Figure S4F). Using a subcutaneous tumor xenograft model, we injected H1975 cells expressing one of two different YES shRNAs or scramble control subcutaneously into nude mice. Three weeks after cell injection, tumors formed with YES knock down cells exhibited significantly reduced tumor volume as well as tumor weight (Figure 5E and 5F). These data suggested that EGFR-mediated YAP1 expression was regulated by YES and that YES is essential for tumor growth in EGFR-dependent lung adenocarcinomas.

### EGFR-dependent cells are sensitive to YAP1 inhibitors

**Figure 6:**
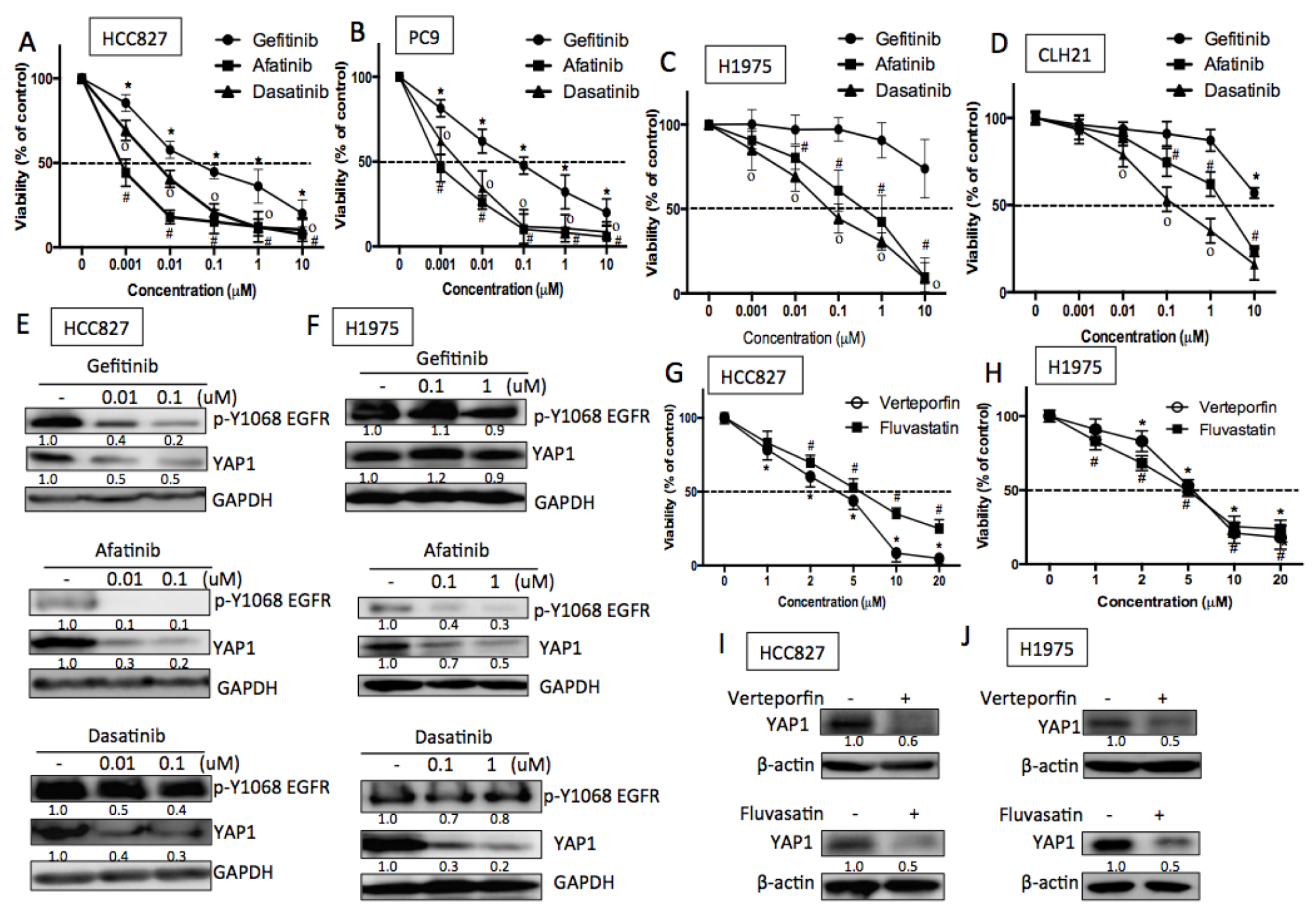
EGFR-dependent cells were sensitive to YAP1 inhibitors **A.** HCC827 and **B.** PC9 cells (exon 19 deletion) were sensitive to EGFR TKIs, afatinib and gefitinib, and were also sensitive to dasatinib. **C.** H1975 (L858R and T790M) and **D.** human primary culture CLH21 (L858R and T790M) cells were insensitive to gefitinib due to T790M but were sensitive to afatinib and dasatinib. The error bar represents the S.E. (*n* = 3). *P < 0.05 vehicle control compared with gefitinib; #P<0.05 compared with afatinib and *P < 0.05 compared with dasatinib treatment. **E.** and **F.** Gefitinib and afatinib reduced YAP1 expression in an EGFR-dependent manner in HCC827 cells, while gefitinib had little effects on H1975 cells EGFR phosphorylation and YAP1 expression. Dasatinib reduced YAP1 level in an EGFR-independent manner in both HCC827 and H1975 cells. EGFR-dependent cells were also sensitive to YAP1 inhibitors, verteporfin or fluvastatin with reduced cell viability in **G.** HCC827 or **H.** H1975 cells. The error bar represents the S.E. (*n* = 3). *P < 0.05 vehicle control compared with verteporfin; #*P* < 0.05 compared with fluvastatin. Reduced YAP1 protein expressions in the presence of verteporfin (1 μM) or fluvastatin (1 μM) were detected in **I.** HCC827 and **J.** H1975 cells 48 h after treatment.

The important roles of YAP1/YES in EGFR-mutant lung adenocarcinoma cells make it a potential therapeutic target for EGFR-dependent cells. Due to the limitation of specific YES inhibitor, a SRC family kinase inhibitor, dasatinib, was used to test the efficacy of YAP1 inhibition in EGFR-dependent cells. HCC827 and PC9 cells that were sensitive to EGFR-TKIs, both gefitinib and afatinib, were also sensitive to the treatment of dasatinib (Figures 6A and 6B). As expected, the two EGFR-TKIs inhibited EGFR phosphorylation as well as YAP1 levels in HCC827 cells. Dasatinib, though had little effect on EGFR phosphorylation, significantly reduced YAP1 protein levels (Figure 6E) and activity (Figure S5A). On the other hand, H1975 or human primary culture CLH21 cells harboring EGFR T790M that contributed to gefitinib resistance were sensitive to afatinib and dasatinib (Figures 6C and 6D). In the T790M mutant H1975 cells, reduced EGFR phosphorylation and YAP1 levels were detected by afatinib treatment, but not by gefitinib treatment. As expected, dasatinib reduced YAP1 levels without affecting EGFR phosphorylation (Figure 6F). According to these data, we assumed that the YAP1/YES pathway is an effective target for EGFR-dependent cells, including EGFR T790M mutation, the most frequent cause of TKI-resistance.

Besides the EGFR-YES-YAP1 axis, YAP1 can be regulated by a variety of mechanisms. We therefore were interested in whether YAP1 inhibitions through other mechanisms can also affect EGFR-dependent lung adenocarcinoma cells. Verteporfin, a photosensitizer, known to reduce YAP1 activity by inhibiting the binding of YAP1 to TEAD, [24] effectively reduced EGFR-dependent cell viability in HCC827 and H1975 (Figure 6G and 6H). YAP1 expression and activity were decreased in the presence of verteporfin detected by immunoblotting and luciferase activity (Figures 6I, 6J and S5B). Statin, a prevalently used lipid lowering reagent, has been reported to act as a YAP1 inhibitor through the inhibition of mevalonate pathway. [25] EGFR-dependent cells were efficiently killed in the presence of statin (Figure 6G and 6H) along with reduced YAP1 protein (Figure 6I & 6J) and target gene expressions (Figure S5C). Dasatinib, verteporfin and fluvasatin, all the three YAP1 inhibitors targeting via different mechanisms effectively reduced viability in EGFR-dependent lung adenocarcinoma cells.

## DISCUSSION

This study demonstrated that YAP1 is an important EGFR downstream signaling molecule that is crucial for lung cancer cell proliferation. A protein in the SRC family, YES, is involved in the regulation of YAP1 by EGFR signaling. By targeting this signaling using a SRC inhibitor, dasatinib, EGFR-dependent cells were effectively killed. Verteporfin and fluvastatin, inhibiting YAP1 activity through EGFR-independnet mechanisms, also reduced cell viability in EGFR-dependent cells. Our data provide evidence that YAP1 can act as a promising therapeutic target for EGFR-depenedent lung adenocarcinoma.

Gefitinib and afatinib are drugs specifically targeting the tyrosine domain of EGFR that inhibit the function of EGFR and cause tumor cell death. Although lung adenocarcinoma patients with EGFR mutations benefit from TKI treatment, they acquire drug resistance after approximately 9 months. A secondary EGFR mutation, T790M, is the most frequent cause of resistance. Although afatinib shows strong beneficial effects on killing EGFR-mutant cells with T790M *in vitro*, its response rate in patients with T790M is not satisfactory.[8] Next-generation EGFR inhibitors, AZD9291, CO-1686, WZ4002 and HM61713 were developed to fight against this most frequent cause of TKI-resistance. Though AZD9291 has recently been approved, acquired resistance with EGFR C797S been reported. [9] Instead of focusing on EGFR itself, here we demonstrate YAP1, an important molecule downstream of EGFR, can act as a potential alternative therapeutic target for EGFR-dependent lung adenocarcinomas, including those with T790M.

In the human lung adenocarcinoma tissue sample analysis, a correlation between EGFR mutation status and YAP1 expression was detected. Though high YAP1-positive rate (59/90) was noted in EGFR mutant group, the YAP1-positive rate was not low (36/74) in EGFR wild-type group. According to our *in vitro* findings, the presence of EGFR ligands that in turn activate EGFR signaling and stabilize YAP1 may explain this phenomenon. Tumor-associated macrophages can secret EGF to the microenvironment [26] that promotes tumor growth, can play an important role in the regulation of YAP1 expression. Unlike the EGFR mutant cells that constitutively autophosphorylates EGFR and promotes YAP1 levels, ligand binding is necessary for EGFR wild-type cells to activate this pathway. Although the mechanisms and physiologic effects of mutant-EGFR and ligand-induced EGFR signaling are not exactly the same, [2] their ability to stabilize YAP1 seems similar in this work.

All the cancer cells accumulate certain mutations in their genome. Some of them are drivers and some are irrelevant. In this study, we demonstrated YAP1 expression in ten cell lines (5 EGFR wild-type and 5 mutant) from different background. All the lines are regulated by various alterations. RASSF1A hypermethylation, detected in H1299 and A549, [27] is known to induce YAP1 activation. [28] However, H1299 and A549 did not show high YAP expression compared to EGFR mutant lines. This gives us an idea that EGFR has stronger effect on YAP1 activation compared to RASSF1A.

YAP1, an effector of Hippo signaling, has emerged as a crucial player in the proliferation and survival of cancer cells. YAP1 was first identified as an oncogene in liver cancer in which YAP1 cooperates with a co-amplified gene cIAP1 to accelerate tumor formation and sustain rapid tumor growth.[13] In β-catenin-driven colon cancer, YAP1 forms a complex with β-catenin and TBX5, which contributes to malignant transformation and promotes colon cancer cell proliferation.[29] In esophageal cancer, YAP1 mediates EGFR overexpression that confers cancer cells chemoresistance.[30]

Although most studies indicate YAP1 acts as an oncogene in different types of cancers, data also suggest that YAP1 has tumor suppressor functions in certain contexts. [31] Yuan et al first reported the tumor suppression role of YAP1 in breast cancer. The detailed mechanisms can be later explained by a series of studies focusing on the crosstalk between Hippo and WNT signaling. YAP1 recruits β-TRCP to the β-catenin destruction complex in the absence of WNT, and thus, reduce the cytoplasm β-catenin retention.[32] Also, cytoplasmic YAP1 suppresses WNT signaling by sequestering Dishevelled protein, that facilitates the WNT signaling transcriptional response. [33] Loss of YAP1 can lead to WNT hypersensitivity with stem cell expansion. [34] Silenced YAP1 is observed in a subset of highly aggressive and undifferentiated colorectal cancer. However, the tumor suppressive role of YAP1 is not detected in our materials of NSCLC.

In a *Drosophila* model, Reddy et al first demonstrated the regulation of YAP1 by EGFR signaling through inhibited Hippo and Ras-MAPK signaling.[35] Here, we demonstrated another signaling pathway linking EGFR and YAP1 instead of the Ras-MAPK pathway. The SRC family protein YES, containing SH2 and SH3 domains, is responsible for the transmission of EGFR signaling to YAP1. The SH2 domain of YES recognizes the tyrosine kinase domain of EGFR and regulates the kinase activity; the SH3 domain of YES non-covalently interacts with YAP1 [36] and may result in the modulation of its kinase activity. Because it is known that YES phosphorylates YAP1 Y357 and thus, stabilizes YAP1, [20, 29] we therefore expected a YAP1 Y357 phosphorylation upon EGFR activation. However, we failed to detect the YAP1 tyrosine phosphorylation using either immunoprecipitated YAP1 with phospho-tyrosine blotting or direct blotting with a phospho-YAP1 (pY357) antibody (Sigma Y0771) in either EGFR active-mutant cells or EGFR wild-type cells treating EGF. The failure to detect YAP1 Y357 phosphorylation upon EGFR activation suggests the existence of routs other than Y357 that regulates YAP1 stability by YES signal.

YAP1 has been identified as a key determinant to enhance treatment sensitivity to EGFR-targeted therapy in lung cancer. [37, 38]. Here in this study, we provided more evidence regarding to the correlation between EGFR activation and YAP1 expression, including human clinical samples. Not only the causal effects, we also had solid mechanistic exploration including the YAP1 stability and β-TRCP binding in the presence or absence of EGFR activation. Moreover, our results demonstrating the role of YES linking EGFR to YAP1 that makes the story more complete. By reducing YAP1 level, we bypassed the EGFR signal and promoted EGFR mutant cell death.

YAP1 is known to be regulated by Rho GTPase in different aspects of studies, for example in stem cell expansion [39] and in mechanotransduction. [16] On the other hand, early studies have revealed the connections between EGFR and Rho GTPase. EGFR mediated cytoskeletal rearrangement dependent on Rho GTPase through Src. [40, 41] According to these data, a potential link of Rho GTPase can be further studied to connect the regulation of YAP1 by EGFR signaling.

With the widespread involvement of YAP1 activation in different cancers, drugs blocking YAP1 activity through different mechanisms can be promising cancer therapeutics. Small molecules inhibiting YAP1 nuclear localization,[42] YAP1-TEAD interactions [24] or activating G protein coupling receptors [43] have been reported to regulate YAP1 activity successfully. A database linking drug data to genomic information has identified dasatinib and statin as a strategy to inhibit YAP1 in cancer cells.[44] Dasatinib, an FDA-approved drug for certain types and conditions of leukemia, has been reported to induce apoptosis in EGFR-dependent lung cancer cells.[45] Phase I/II and phase II clinical trials using dasatinib to treat NSCLC patients have been performed without specific selection and showed only modest effects. [46–48] In consistent with previous study, our work demonstrate efficacy of dasatinib in lung adenocarcinoma cells with EGFR-dependnecy via inhibition of YAP1 signaling. Our results may provide indications regarding patient selection for the use of dasatinib in lung cancer therapy.

Moreover, statins, long and prevalently used as a lipid-lowering agent for patients with hyperlipidemia, have been reported to be associated with reduced cancer-specific mortality. [49] A cohort study with British database [50] has demonstrated reduced rates of cancer-specific mortality in lung cancer patients with regular statin use. The YAP1 inhibiting properties of statins and its ability to kill EGFR-mutant lung adenocarcinoma cells may provide some evidence explaining this interesting phenomenon.

In this study, we have addressed the importance of YAP1 in lung adenocarcinomas by identifying YAP1 as an important EGFR downstream mediator regulating cell growth. By reducing YAP1 activity through different mechamnisms, EGFR-dependent lung adenocarcinoma cells were effectively killed. This study provides evidence that YAP1 can act as a promising alternative therapeutic target in patients with EGFR-mutant lung adenocarcinoma, including those with EGFR T790M.

## MATERIALS AND METHODS

### Tissue samples

A total of 164 cases of lung adenocarcinoma paraffin-embedded tissue were obtained from the Pathology Department of Taipei Veterans General Hospital. Material was approved by the Institutional Review Board of the Taipei Veterans General Hospital. EGFR mutation status was detected using PCR method.[18] For Immunohistochemistry staining of YAP1, tissue samples were retrieved by retrieval buffer (Dako, #8005) and staining was performed using YAP1 antibody (Cell signaling, #4912).

### Cells and cell culture

The following human lung cancer cell lines were used in this study: A549 (EGFR wild-type); H1975 (EGFR L858R and T790M mutations); HCC827 (EGFR exon 19 deletion) ; and PC9 (EGFR exon 19 deletion) cells. Human embryonic kidney cells HEK 293T were used to produce viruses for knocking down genes using shRNAs. A549, H1975, HCC827 and HEK293T cells were obtained from the American Type Culture Collection during 2012. Cell authentication was last performed by STR profiling in July 2014. To compare YAP1 expression levels in EGFR wild-type versus EGFR mutant cells, 5 EGFR wild-type (Hop62, H358, H1299, CL1-0 and A549) and 5 EGFR mutant NSCLC cell lines (HCC827, H1975, PC9, H1650 and H820) were collected 4 h post serum starvation. CLH21, a primary culture of tumor cells from a lung adenocarcinoma patient with EGFR-TKI resistant due to secondary EGFR T790M mutation was harvested from malignant pleural effusion of a patient who was refractory to EGFR-TKI at the National Taiwan University Hospital (NTUH). The lung cancer cells were grown in RPMI-1640 with 10% fetal bovine serum and cultured at 37 °C in a humidified incubator.

### Plasmids

The YAP1-responsive synthetic promoter driving the luciferase plasmid 8xGTIIC-Luc (Addgene 34615) was obtained from Addgene.

### Proliferation assay

H1975 or HCC827 cells (10^3^/well) with shControl, shYAP1 or shYES were seeded in 96-well plates. Cell proliferation was measured using AlarmBlue (Invitrogen, Grand Island, NY, USA). A standard curve was created by measuring the signals from different density of cells (from 2,000 to 64,000 cells). Fluorescence of 560 nm excitation and 590 nm emission was measured.

### Reporter assay

For the characterization of TEAD activity in lung cancer cells, the cells were transfected with 8xGTIIC (addgene, #34615) and PRL-TK plasmids. The cells were plated in 6-well plates, and the day after transfection, EGF was administered for 3 h; afatinib, dasatinib, verteporfin or fluvastatin treated for 2 days. Luciferase luminescence was measured using the Dual-Glo luciferase assay kit (Promega, Madison, WI, USA).

### Xenograft tumorigenicity assay

A xenograft assay was performed in 4-week-old male BALB/c nude mice. H1975 cells infected with scramble control, shYAP1 or shYES plasmids were collected. A total of 10^6^ cells in PBS mixed with an equal amount of Matrigel were injected subcutaneously into the right and left side of the flank region. All animal experiments were performed in accordance with the animal guidelines of the Acdemia Sinica Institute Animal Care and approved by the Animal Care Committee.

### Viability assay

Cells (3×10^3^/well) seeded in 96-well plates were treated with different concentrations of gefitinib, afatinib, dasatinib or verteporfin or fluvastatin for 72 h. Cell viability was detected using the MTT reagent, and absorbance at 540 nm was measured.

## SUPPLEMENTARY MATERIALS FIGURES AND TABLES


