# Supplementary material for: YAP1 is essential for tumor growth and is a potential therapeutic target for EGFR-dependent lung adenocarcinomas
Source: Oncotarget. 2017 Jul 27;8(52):89539–51. doi: 10.18632/oncotarget.19647 (PMC5685690; doi:10.18632/oncotarget.19647)
Supplement: Supplementary file 1 [file oncotarget-08-89539-s001.pdf]

YAP1 is essential for tumor growth and is a potential therapeutic target for EGFR-dependent lung adenocarcinomas

SUPPLEMENTARY MATERIALS

Chemicals

Recombinant human EGF (30 ng/ml) and TGF-α (10 ng/ml; PeproTech, Rocky Hill, NJ, USA) are EGFR ligands used to activate EGFR signaling. MG132 (10 μM), cycloheximide (CHX, 20 μg/ml), verteporfin and fluvastatin were purchased from Sigma (St. Louis, MO, USA). Afatinib, gefitinib and dasatinib (Selleckchem, Houston, TX, USA) 0.001 to 10 μM were used to investigate cell viability. MG132, cycloheximide, verteporfin, fluvastatin, afatinib, gefitinib and dasatinib were all dissolved in dimethyl sulfoxide (DMSO) with a final concentration of DMSO < 0.1 %. The vehicle control group contained 0.1 % DMSO.

shRNA expression

Lentivirus containing short hairpin RNAs (shRNAs) expressed in a lentiviral vector (pLKO.1-puro) were generated in 293 T cells. Various pLKO plasmids to knockdown EGFR, YAP1, YES, and scrambled control were provided by National RNAi Core Facility of Academia 1Sinica, Taipei, Taiwan. For lentivirus production, 293T cells in 6-cm dish were transfected with 2.5 μg pLKO.1-puro lentiviral vectors expressing different shRNAs along with 0.25 μg of envelope plasmid pVSVg and 2.5 μg of packaging plasmid pCMVΔR8.91. Virus was collected 48 h after transfection. To prepare various knockdown cells, H1975, HCC827 or A549 cells were infected with lentivirus for 24 h, and followed with 2 μg/ml puromycin selection. The sequences of the lentiviral shRNAs were shown in Table S1.

Immunoblotting

Cells were lysed using RIPA buffer with the addition of proteases and phosphatases inhibitors (Roche, Indianapolis, IN, USA). Quantified lysates were separated on an SDS polyacrylamide gel. The blots were probed with the indicated antibodies as follows: EGFR, phospho-EGFR (Y1068), YAP1, phospho-YAP (S127), YES and β-TRCP (all from Cell Signaling, Beverly, MA, USA); and actin (Sigma).

Quantitative RT-PCR

RNA was harvested from cells using Trizol. Complementary DNA was prepared using Transcriptor Reverse Transcriptase (Roche). Quantitative PCR was performed using SYBR Green (Applied Biosystems, Foster City, CA, USA). The primers used are listed in Table S2.

Co-immunoprecipitation assay

Lysis buffer [50 mM Tris-HCl pH 7.5, 150 mM NaCl, 1 mM DTT, 1% NP-40 (v/v) containing protease inhibitors and phosphatase inhibitors] was added to the cells. The lysates were incubated with YAP1 (Cell Signaling) or EGFR (MA5-13697, Thermo) antibodies overnight at 4 °C. The lysates were then incubated with Dynabeads protein G (Invitrogen) for 1 h at 4 °C and washed twice with wash buffer [50 mM Tris-HCl pH=7.5, 150 mM NaCl, 1 mM DTT, 1% NP-40 (v/v)].

Immunofluorescent staining

The lung cancer cell line cells were plated on a microscope slide and fixed using 4% paraformaldehyde 24 h after plating. Cells were permeabilized by adding 0.1% Triton in PBS for 5 min. Cells were incubated with 1% BSA in PBS containing 1:100 anti-YAP1, YES or EGFR (Cell Signaling) overnight at 4 °C. Mouse or rabbit fluorescent secondary antibody was added (1:500) for 1 h at RT.

Statistical analyses

The data were analyzed using Student's t test or one-way ANOVA. P values <0.05 were considered significant. The IHC results comparing the correlation between EGFR mutation and YAP1 expression was performed using Fisher's exact test.

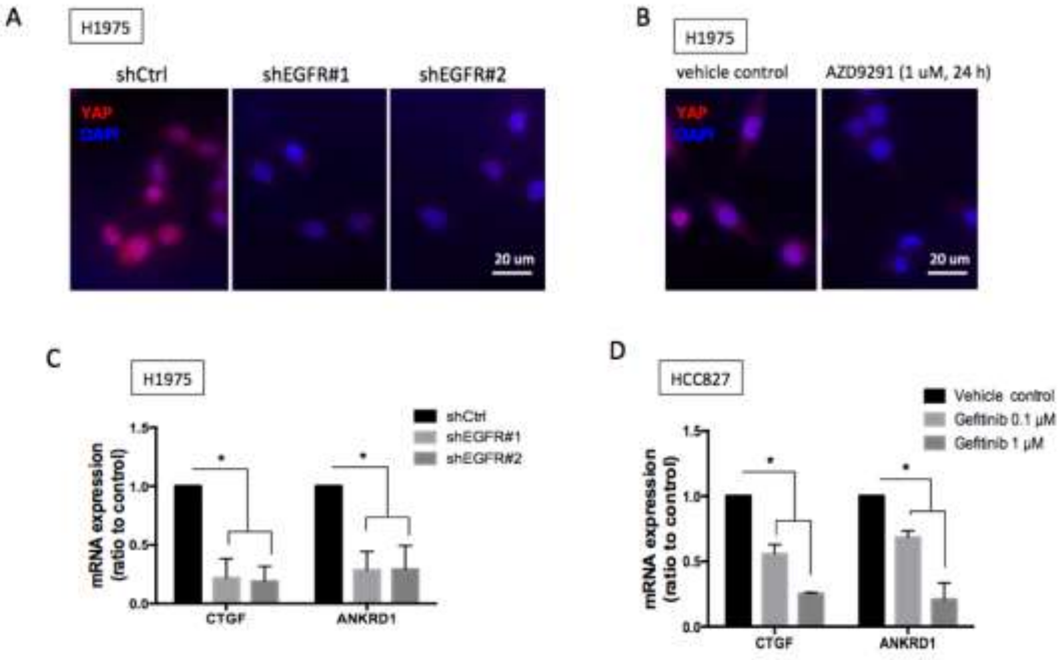

Figure S1. EGFR inhibition suppressed YAP1 activity. Immunocytochemistry demonstrated reduced YAP1 expression in H1975 cells in (A) EGFR knocked down groups and (B) in the presence of 3rd generation TKI, AZD9291 (1 µM, 24 h). Reduced CTGF and ANKRD1 mRNA expression detected in (C) H1975 EGFR knockdowns and (D) HCC827 cells in the presence of gefitinib (24 h).

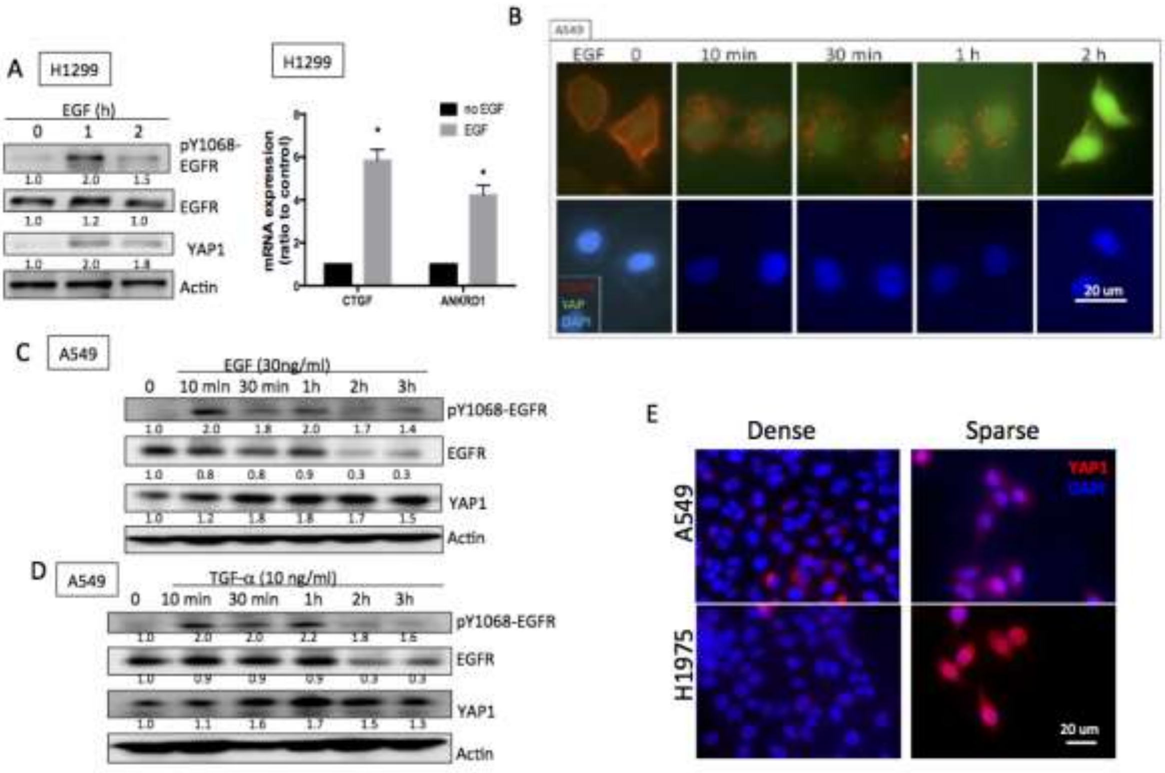

Figure S2. EGFR signaling regulated YAP1 protein level. (A) Enhanced YAP1 protein level and target gene mRNA in response to EGF stimulation in H1299 cells. (B) In A549 cells, YAP1 was weakly expressed and EGFR was located around cell membrane without EGF treatment. EGFR internalization was detected along with the strong YAP1 nuclear localization in the presence of EGF. (C & D) The two EGFR ligands, EGF and TGF-α (10 min to 3 h) both induced EGFR phosphorylation as well as YAP1 accumulation. (E) The EGFR active-mutant lung adenocarcinoma cell lines H1975 and HCC827 showed stronger YAP1 signal and nuclear localization compared to EGFR wild type A549 detected by immunocytostaining.

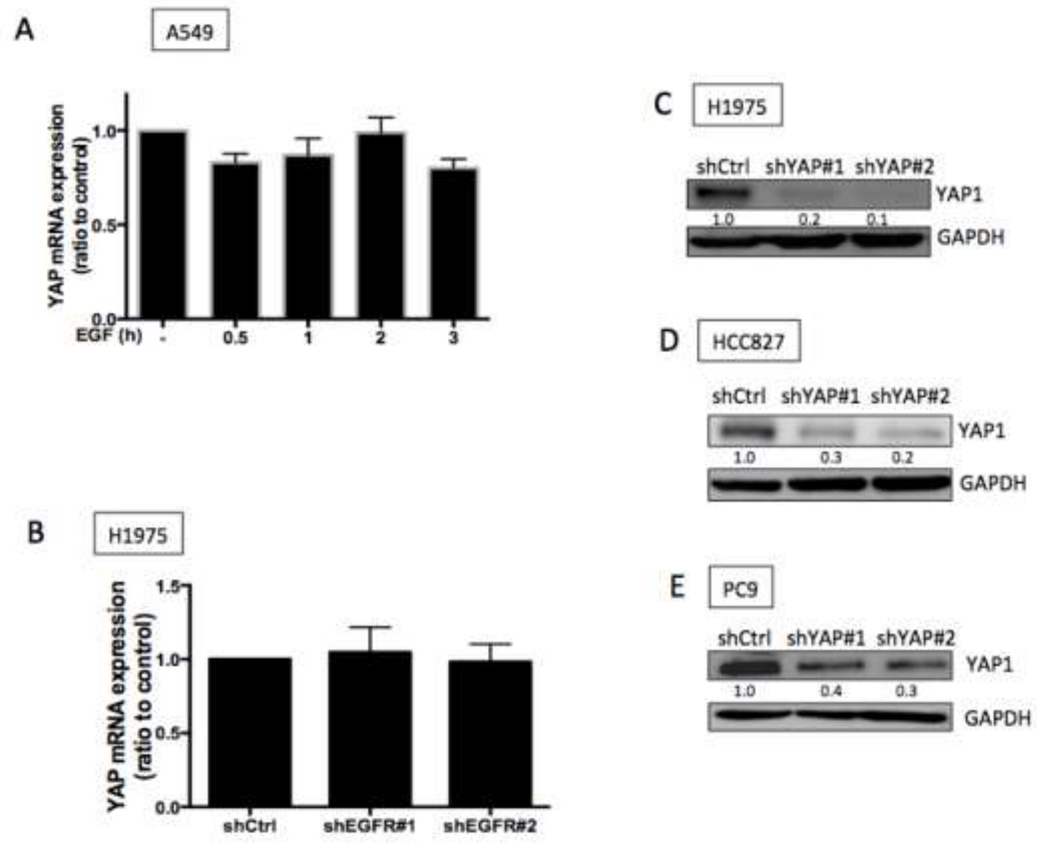

Figure S3. EGFR signaling had no effects on the regulation of YAP1 mRNA levels. (A) In A549 cells, YAP1 mRNA levels were not regulated with EGF treatment. (B) YAP1 mRNA levels were not affected in EGFR knockdown H1975 cells. (C-E) Reduced YAP1 protein expression in YAP1 knockdowns using shYAP1 in H1975, HCC827 and PC9 cells.

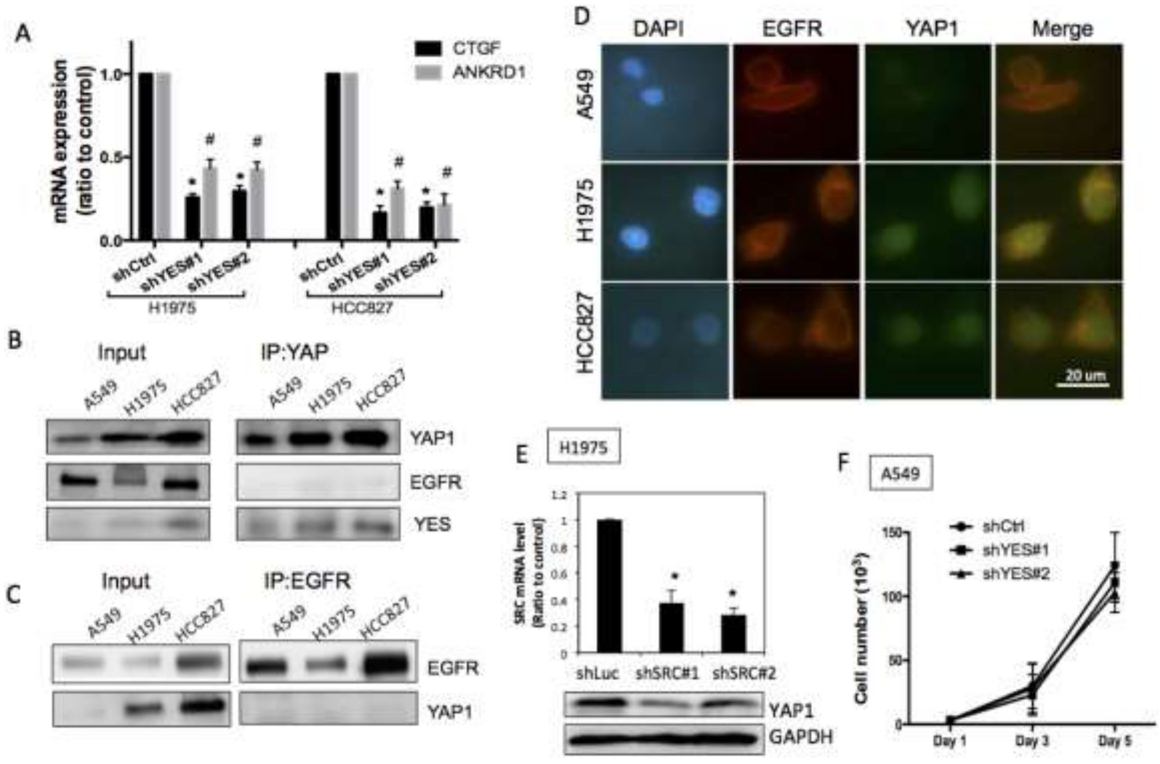

Figure S4. Interactions between EGFR, YES and YAP1. (A) Reduced target genes, *CTGF* and *ANKRD1*, expression in the knockdowns of YES in both H1975 and HCC827 cells. (B) By immunoprecipitating YAP1, its binding with EGFR was not detected; yet the binding with YES was detected in A549, H1975 and HCC827 cells. (C) By immunoprecipitating EGFR, its binding with YAP1 was not detected. (D) Double immunostaining of EGFR and YAP1. Colocalization of EGFR and YAP1 was not detected. (E) In H1975 cells, reduced YAP1 expression was detected in SRC knockdowns using shSRCs. (F) No significant effect on cell proliferation was detected in A549 shYES cells.

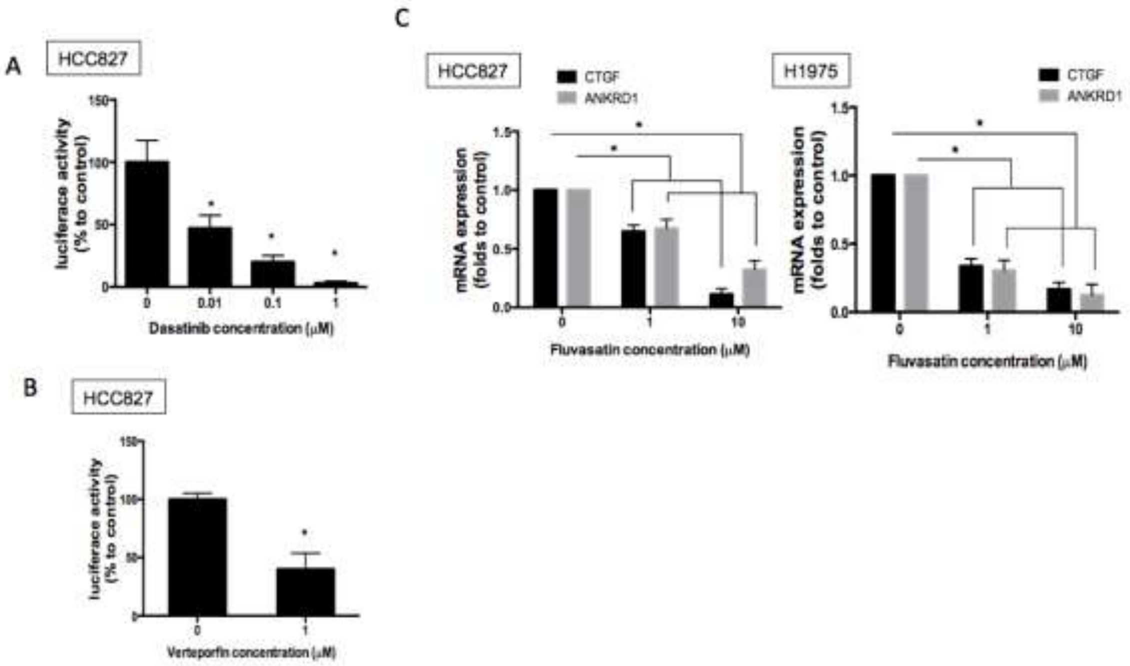

Figure S5. Dasatinib, verteporfin or fluvastatin reduced YAP1 activity. (A) Dasatinib and (B) verteprofin significantly reduced YAP1 activity by reducing the 8XGTIIC luciferase activity. (C) Fluvastatin significantly reduced target genes, *CTGF* and *ANKRD1*, expression in both H1975 and HCC827 cells.

Table S1. Sequence of the lentiviral shRNAs

**Target gene**	Sequence
EGFR	GCCAAGCCAAATGGCATCTTT; GCCACAAAGCAGTGAATTTAT
YAP1	CAGGTGATACTATCAACCAAA; CCCAGTTAAATGTTCACCAAT
YES	TGGTTATATCCCGAGCAATTA; GCTGCACTGTATGGTCGGTTT
SRC	GACAGACCTGTCCTTCAAGAA; GCGGCTCCAGATTGTCAACAA

Table S2. Sequences of QPCR primers

**Gene**	**Forward**	**Reverse**
**GAPDH**	GCATTGCCCTCAACGAC	GTCTCTCTCTTCCTCTTGTGC
**YAP1**	CTCCCCAGTGACGAGAGAGC	CTAGGTCTGCGACCTCGAC
**CTGF**	CCTGGTCCAGACCACAGAGT	TGGAGATTTTGGGAGTACGG
**ANKRD1**	AGAACTGTGCTGGGAAGACG	GCCATGCCTTCAAAATGCCA
